# The Role of Interspecific Hybridisation in Adaptation and Speciation: Insights From Studies in *Senecio*

**DOI:** 10.3389/fpls.2022.907363

**Published:** 2022-06-23

**Authors:** Edgar L. Y. Wong, Simon J. Hiscock, Dmitry A. Filatov

**Affiliations:** ^1^Department of Plant Sciences, University of Oxford, Oxford, United Kingdom; ^2^Oxford Botanic Garden and Arboretum, Oxford, United Kingdom

**Keywords:** hybridisation, speciation, adaptation, *Senecio aethnensis*, *Senecio chrysanthemifolius*, Mount Etna

## Abstract

Hybridisation is well documented in many species, especially plants. Although hybrid populations might be short-lived and do not evolve into new lineages, hybridisaiton could lead to evolutionary novelty, promoting adaptation and speciation. The genus *Senecio* (Asteraceae) has been actively used to unravel the role of hybridisation in adaptation and speciation. In this article, we first briefly describe the process of hybridisation and the state of hybridisation research over the years. We then discuss various roles of hybridisation in plant adaptation and speciation illustrated with examples from different *Senecio* species, but also mention other groups of organisms whenever necessary. In particular, we focus on the genomic and transcriptomic consequences of hybridisation, as well as the ecological and physiological aspects from the hybrids’ point of view. Overall, this article aims to showcase the roles of hybridisation in speciation and adaptation, and the research potential of *Senecio*, which is part of the ecologically and economically important family, Asteraceae.

## Introduction

Understanding the evolutionary genetic processes that underpin phenotypic adaptation and speciation is fundamental for understanding the process of Darwinian evolution. It has been more than 160 years since Darwin described how species adapt and evolve through the force of natural selection, but despite the subsequent advances in population genetics and evolutionary theory, our understanding of adaptation and speciation is still far from complete (Coyne and Orr, 2004; Rieseberg and Willis, 2007; Abbott et al., 2009). Speciation is one of the oldest problems in evolutionary biology, which has successfully resisted the efforts of generations of evolutionary biologists (e.g., Coyne and Orr, 1989). The advance in molecular genetics techniques in the last 15 years or so resulted in the reincarnation of the field which became one of the hottest topics of evolutionary biology (e.g., Ravinet et al., 2017; Campbell et al., 2018; Becraft and Moya, 2021). The role of interspecific hybridisation in adaptation and speciation is actively debated in the literature and its importance becomes more apparent (e.g., Ebersbach et al., 2020; Nevado et al., 2020; Wong et al., 2020; Hobbs et al., 2021; Bush, 2022).

Plant speciation (or at least the literature on plant speciation) differs substantially from that in animals. Plant literature often focuses on species hybridisation and introgression during speciation, rather than on reproductive isolation (reviewed in Abbott, 1992). Historically, the animal-focused researchers considered hybridisation an evolutionary dead-end (Mayr, 1963) since it homogenises the diverging genomes and prevents speciation. However, plant biologists (Anderson, 1948; Anderson and Stebbins, 1954; Grant, 1972) have long considered hybridisation as an important force in adaptation and speciation. Indeed, hybridisation is widespread in plants (e.g., Grant, 1972; Mallet, 2001; Rieseberg et al., 2004), and it may play a substantial role in the adaptation and speciation of plant populations (Barton, 2001; Rieseberg et al., 2003). Recent studies have shown that hybridisation can have more complex outcomes than just homogenisation of diverging genomes. For example, it could lead to extinction of hybrid lineages, evolution of new species (hybrid speciation), and introgression of adaptive alleles, leading to faster adaptation. While the importance of interspecific hybridisation in evolution is becoming more apparent, the extent (and the role) of gene exchange during hybridisation of plant and animal species is not entirely clear.

Many partially isolated species are known to form hybrid zones. A *Helianthus* hybrid zone was demonstrated to be ‘semi-permeable’, meaning that while there was a barrier to gene flow of some genomic regions, the majority of the genome can introgress freely (Rieseberg et al., 1999). On the other hand, the two *Senecio* species forming an elevational hybrid zone on Mount Etna, Sicily, are fully compatible, though the evidence for numerous interspecific incompatibilities between these species is starting to emerge (Brennan et al., 2014, 2019; Chapman et al., 2016). Hybrid zones can be considered as ‘windows on the evolutionary process’ (Harrison, 1993) and they represent ‘evolutionary laboratories’ providing the researchers an opportunity to analyse and dissect the role of hybridisation in speciation and adaptation. In particular, the analyses of hybrid zones inform the debate whether hybrids are an evolutionary dead-end (Mayr, 1963) or play a more creative role in adaptation and speciation (Anderson, 1948; Arnold, 1997; Rieseberg, 1997).

There are several ways in which hybridisation could promote speciation and adaptation (Seehausen, 2013; Vallejo-Marín and Hiscock, 2016). Hybridisation can either act to transfer adaptive alleles between lineages to aid adaptation, or result in hybrid speciation with or without polyploidisation. Allopolyploid hybrid speciation occurs when two parental lineages with different chromosome ploidies hybridise. This may result in hybrids with an odd (often sterile) or even number of chromosome sets. Some of the sterile hybrid populations may persist without reproduction due to constant hybridisation events, while some can reproduce asexually. Others may undergo further genome duplications to overcome genomic conflicts such as chromosomal pairing during meiosis (e.g., *Mimulus peregrinus*; Vallejo-Marín, 2012; Vallejo-Marín et al., 2015). Homoploid hybrid speciation occurs when both parental lineages have the same chromosome number (e.g., Italian Sparrow, *Passer iltaliae*; Hermansen et al., 2011; and Oxford ragwort, *Senecio squalidus*; James and Abbott, 2005). Depending on their origin and genomic structure, hybrids have different obstacles to overcome (such as problems in meiosis and gene regulation) and different evolutionary pathways to eventually become a reproductively isolated taxon. Even if hybridisation does not lead to speciation, it can provide opportunities for adaptation. Following hybridisation, hybrid lineages often experience tremendous changes compared to their parents. Instead of detailing all the consequences of hybridisation on the phenotypic and genomic level, this short review focuses on the ones that are potentially beneficial for adaptation and speciation. Examples from different *Senecio* species will be used to illustrate the role of hybridisation in adaptation and speciation.

*Senecio* L. is a genus of herbaceous plants, shrubs, small trees and climbers in the Asteraceae family. The genus has a worldwide distribution, containing at least 1,400 described species (Royal Botanic Gardens Kew, 2022), many of which are cultivated extensively. Alongside other genera such as *Artemisia*, *Cynara*, *Echinacea*, *Helianthus*, *Lactuca*, *Tragopogon*, the Asteraceae family presents huge economic values, with numerous species being used in food, medicine, and horticulture. The ‘*Senecio* system’ is also rapidly becoming recognised as one of the most tractable plant models in which to study the process of speciation at a genetic, genomic, and ecological level (Abbott and Rieseberg, 2012; Gross, 2012; Walter et al., 2020). The fact that speciation events in the genus have occurred relatively recently, and involve examples of both ecological speciation and hybrid speciation (homoploid and allopolyploid; Abbott and Rieseberg, 2012; Hegarty et al., 2012), make *Senecio* a unique alternative to more conventional plant models, such as *Arabidopsis*, for studies of plant evolution in action.

**Figure 1:**
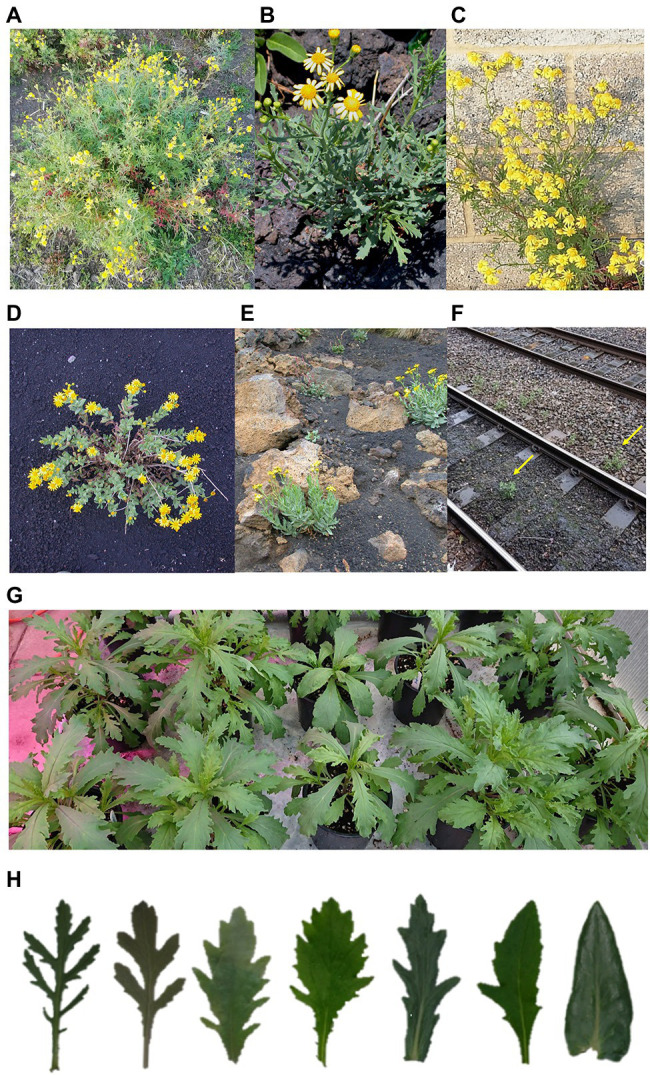
Examples of phenotypes and habitats of *Senecio aethnensis*, *Senecio chrysanthemifolius*, their natural hybrids and *Senecio squalidus*. **(A)** *S. chrysanthemifolius* from Mount Etna. **(B)** Natural hybrid on Mount Etna. **(C)** Homoploid hybrid species *S. squalidus* from Oxford, United Kingdom. **(D)** *S. aethnensis* from Mount Etna. **(E)** Natural habitat of *Senecio* on Mount Etna. **(F)** *S. squalidus* found on railways in the United Kingdom (identified by yellow arrows). **(G)** Variation in leaf phenotypes in one F_2_ synthetic hybrid (between *S. aethnensis* and *S. chrysanthemifolius*) family. **(H)** Leaf shape variation in greenhouse-grown *Senecio*. Left most: *S. chrysanthemifolius*; Right most: *S. aethnensis*; All in-between: synthetic F_2_ hybrids.

Natural hybridisation and stable hybrid zones present natural experiments that can be dissected at the molecular level to identify genomic factors associated with local adaptation and the maintenance of species differences and boundaries (Grant, 1981; Rieseberg, 1997; Arnold, 2006; Lexer and Widmer, 2008). A classic example of natural hybridisation is found on Mount Etna, Sicily. Here, two species of *Senecio*, *S. aethnensis* and *S. chrysanthemifolius*, which are locally adapted to high- and low-elevation conditions respectively, form a stable hybrid zone at the boundaries of their respective ecological ranges mid-way up the volcano. *S. aethnensis* populations are found at high elevations [>2,000 meters above sea level (masl)] and *S. chrysanthemifolius* are found at low elevations (<1,000 masl; Brennan et al., 2009; Muir et al., 2013). The two species are distinguishable through an array of phenotypic (such as leaf dissection: Figure 1; James and Abbott, 2005; Brennan et al., 2009; Wong et al., 2020), physiological (such as seed germination temperature: Ross, 2010), and ecological differences (such as flowering time). Significant differences between these species have also been observed at the level of gene expression (Hegarty et al., 2009; Muir et al., 2013; Chapman et al., 2016), and candidate genes identified in these studies are predicted to be adaptive (Wong et al., 2020). For instance, genes predicted to be involved in adaptation to high light intensity, UV stress, sulphur metabolism, dehydration and cold stress are highly expressed in *S. aethnensis* compared to *S. chrysanthemifolius* (Hegarty et al., 2009). The two species maintain a hybrid zone at intermediate elevations, where hybrids display intermediate phenotypes (James and Abbott, 2005; Brennan et al., 2009). Despite recent divergence (<200 KYA; Chapman et al., 2013; Muir et al., 2013; Osborne et al., 2013) and regular gene flow, *S. aethnensis* and *S. chrysanthemifolius* evolved as distinct species and maintain very different phenotypes, with leaf shape showing the most extreme differences at the phenotypic level (Figure 1). How the species identity is maintained despite the on-going gene flow remains unclear, but it was suggested that multiple factors act together to keep the species identity, including genetic incompatibilities (Brennan et al., 2014, 2016, 2019; Chapman et al., 2016), strong divergent selection and selection against hybrids (Brennan et al., 2009; Wong et al., 2020). For instance, transmission ratio distortion (TRD) was also identified in this system (34 out of 127 marker loci in Brennan et al., 2014; three regions in Chapman et al., 2016; 2.9%–26.8% of loci in Brennan et al., 2019), with pre-zygotic events (such as gametophytic selection), cytonuclear incompatibility, Bateson–Dobzhansky–Muller incompatibility and potentially underdominance contributing to these TRFs (Brennan et al., 2014). Hybrid breakdown as a consequence of genetic incompatibilities was also observed in synthetic hybrids. Some of the breakdown traits include low germination and albinism (Hegarty et al., 2009), necrotic growth (Chapman et al., 2016) and early mortality (Brennan et al., 2014). Thus, current data strongly suggest that *S. aethnensis* and *S. chrysanthemifolius* represent a clear-cut case of ecological speciation driven by adaptation to contrasting conditions of high- and low-elevation. There are relatively few studied cases of ecological speciation (Counterman et al., 2010; Martínez-Fernández et al., 2010; Papadopulos et al., 2011), making *Senecio* a particularly valuable model system for research in adaptation and speciation.

Another attractive feature of this study system is a case of rapid recent (<300 years) homoploid speciation of *S. squalidus* in the United Kingdom. This speciation occurred following the introduction of *Senecio* plants from Mount Etna to England some 300 years ago (Nevado et al., 2020). This case of speciation is relatively well documented because it occurred in Oxford Botanic Garden, hence the common name of *Oxford Ragwort*. Originating from hybridisation an English garden and a period of sustained cultivation in Oxford, *S. squalidus* has now spread to the majority of the United Kingdom as far north as Scotland, and was found to hybridise with native species, such as Groundsel (*Senecio vulgaris*) leading to the origin of two new allopolyploid species, the allohexaploid *S. cambrensis* and the allotetraploid *S. eboracensis* (Lowe and Abbott, 2004). This system presents an exciting model for studying speciation, adaptation, invasion and hybridisation (e.g., Nevado et al., 2020; Walter et al., 2020). It also presents an excellent example of how hybridisation can lead to speciation.

## Roles of Hybridisation in Adaptation and Speciation

### Transcriptome Shock

Studies have shown altered gene expressions in hybrids compared to parental lineages, a process known as ‘transcriptome shock’ (Lee and Chen, 2001; Shaked et al., 2001; Adams et al., 2003; Adams and Wendel, 2005; Comai, 2005; Madlung et al., 2005; term first used in Hegarty et al., 2006). It is worth stressing that although transcriptome shock is often observed in polyploid hybrids, it is an outcome of hybridisation, rather than genome duplication (Wang et al., 2006). The alterations to gene expressions are found to be nonadditive (Wang et al., 2006; Hegarty et al., 2011), immediate in F_1_ hybrids but stable in subsequent hybrid generations (Comai et al., 2000; Adams et al., 2003; Hegarty et al., 2006, 2009; Wang et al., 2006). Studying the triploid hybrids (*S. x baxteri*) between the tetraploid *S. vulgaris* and diploid *S. squalidus*, and hexaploid allopolyploid (*S. cambrensis*) arisen from the triploid hybrid, Hegarty et al. (2006) showed that transcriptome shock was evident in *S. x baxteri* and that the shock was ‘ameliorated’ after genome duplication in the *S. cambrensis*. It could manifest in mechanisms involving gene silencing, regulatory networks, chromatin remodelling and DNA methylation (Shaked et al., 2001; Madlung et al., 2005). Although this epigenetic instability could be disadvantageous, it could serve as a target for selection to act on to subsequently facilitate adaptation and speciation in the hybrid lineage (Hegarty et al., 2011).

In the homoploid hybrid species *S. squalidus*, two genes, ATP-sulfurylase precursor and glutathione-S-transferase, were found to have transgressive up-regulation compared to the midpoint of the parental species (Hegarty et al., 2009). These two genes are likely up-regulated in response to deficiency in sulphur (Xiang and Oliver, 1998; Harada et al., 2002), as most of United Kingdom soils contain much less sulphur (<20 kg/ha/year; Brown et al., 2000) than soil on Mount Etna where the parental species live (>40 kg/ha/year in quiescent period between 1997 and 2001, and much more following volcanic eruption; Aiuppa et al., 2006). Research has shown that the hybridisation event leading to speciation of *S. squalidus* most likely happened after parental plants were brought to the United Kingdom, instead of hybrid material arriving in the United Kingdom from Mount Etna, as thought previously (Nevado et al., 2020). Hence the altered gene expression observed in *S. squalidus* likely evolved due to hybridisation but not pre-adaptation. This is a good example of how transcriptome shock can facilitate adaptation in hybrid lineages in a novel environment which is drastically different than the parental ones (in this case includes sulphur level).

### Genome Reorganisation

It is not uncommon for hybrid lineages to experience genome reorganisation (‘genome shock’: e.g., Rieseberg, 2001; Chen and Ni, 2006), such as chromosomal rearrangements, translocations, and movement of transposable elements. These rearrangements may not be involved in adaptation to new environments, but they often serve as a form of reproductive isolation from parental lineages through restricting backcrossing (Rieseberg et al., 2003; Coghlan et al., 2005; Hegarty and Hiscock, 2005; Paun et al., 2007), an important step in speciation. Genomic restructuring is also commonly observed in new, successful hybrid lineages alongside other ecological and spatial divergence from progenitors (Buerkle et al., 2000; Baack and Rieseberg, 2007; Karrenberg et al., 2007; Brennan et al., 2019), and might be crucial to restore nucleocytoplasmic compatibility (Soltis and Soltis, 1999).

Hybridisation-induced chromosomal rearrangements have been documented in a few allopolyploid species such as *Triticum* (Levy and Feldman, 2004), *Nicotiana* (Lim et al., 2004) and *Arabidopsis* (Pontes et al., 2004); as well as homoploid species such as *Helianthus* (e.g., Burke et al., 2004; Lai et al., 2005), *Iris* (Tang et al., 2010; Taylor et al., 2013), *Agrodiaetus* (Lukhtanov et al., 2015), and our focal group *Senecio* (Brennan et al., 2019).

Comparing the genome structure of *S. squalidus* and its progenitors *S. aethnensis* and *S. chrysanthemifolius* using genetic mapping, it was found that there are indeed differences in genomic architecture between the latter two and this led to the inheritance of some of this genetic incompatibility in *S. squalidus* (Brennan et al., 2019). Comparison between genetic maps of F_2_ mapping families with either parent also revealed genomic reorganisation between maps in half of the linkage groups (Brennan et al., 2019). They also showed evidence for colocation between transmission ratio distortion loci and genomic rearrangements. These rearranged regions were hypothesised to contribute to incompatibilities and reproductive isolation, and where divergent selection acts to promote adaptation and speciation. This hypothesis can be tested with the *S. squalidus* genome that will soon be available.

### Increased Heterozygosity, Heterosis and Transgressive Segregation

Another opportunity for adaptation and speciation in both homoploid and polyploid hybrid lineages is heterosis, in which the hybrid lineages express more vigorous phenotypes compared to parental lineages due to increased heterozygosity; and transgressive segregation, in which extreme phenotypes (positive or negative) are formed. Because of recombination and transgressive effects, hybrids usually possess higher level of variation compared to parental lineages, which creates vast potential for novel evolutionary trajectories (Arnold, 2006; Abbott and Brennan, 2014).

In *Senecio*, heterosis was observed in the F_1_ hybrids between *Senecio jacobaea* and *S. aquaticus*. Hybrids had superior fitness, and they were found to possess adaptations such as drought and flooding resistance, inherited from either parent, respectively, (Kirk et al., 2005). These features would allow the hybrid lineage to expand and occupy niches outside of their parental ones. Unlike homoploid hybrids whose heterozygosity is expected to decline over generations due to recombination, the enforced pairing of homologous chromosomes in polyploid hybrids inhibits intergenomic recombination, thus conserving the high level of heterozygosity through generations (Comai, 2005). An exceptional example of the role of polyploid hybridisation in adaption and speciation is the arctic flora. Research has suggested that polyploid lineages are better at colonising after deglaciation compared to diploid lineages, and that the polyploid lineages’ fixed-heterozygosity prevented the disadvantageous effects of inbreeding and loss of heterozygosity caused by genetic drift (Brochmann et al., 2004).

Transgressive segregation is very commonly applied in crop breeding, but it can also be found in the wild species. For example, the homoploid species, *Helianthus anomalus*, *H. paradoxus*, and *H. deserticola* (all originated from the same pair of parental species), occupy different habitats and also exhibit adaptive traits not seen in the parental species (Schwarzbach and Rieseberg, 2002; Welch and Rieseberg, 2002; Gross et al., 2003; Gross and Rieseberg, 2005; Lai et al., 2005). This demonstrates that hybridisation is able to generate novelty in terms of morphology, anatomy, life history and physiology which in turn allows for adaptation and speciation (Abbott et al., 2010). Transgressive up-regulation of genes were also observed in *Senecio* (discussed above: Hegarty et al., 2009).

### Change in Mating System and Reproductive Traits

Hybridisation and polyploidisation can sometimes lead to a different mating system in the hybrids. For instance, it is well-known that allopolyploidy is frequently associated with a shift from self-incompatibility (in the parental species) to self-compatibility in the hybrid polyploid (Entani et al., 1999; Miller and Venable, 2000; Nasrallah et al., 2000; Brennan and Hiscock, 2010); and be associated with asexual reproduction, both vegetative and agamospermy (Otto and Whitton, 2000; Janko et al., 2012). In the arctic flora, numerous diploid taxa of hybrid origin are self-compatible or clonal, making them successful in the arctic environment where pollinators are scarce (Brochmann et al., 2004). Having a different mating system would allow these hybrid taxa to occupy new niches and/or perpetuate in smaller populations since there is reduced reliance on pollinators and mating partners.

Hybridisation between the tetraploid *S. vulgaris* and diploid *S. squalidus* in the United Kingdom has also resulted in two hybrid species with varying reproductive traits and mating system. *S. vulgaris* is self-compatible with capitula that are rayless; whereas *S. squalidus* is self-incompatible with capitula showing a mix of ray and disc florets. Their hybridisation led to the evolution of an allohexaploid species, *S. cambrensis*, and tetraploid species, *S. eboracensis* (Lowe and Abbott, 2004; Brennan and Hiscock, 2010; Hegarty et al., 2012). Both hybrid species possess self-compatibility of *S. vulgaris* and ray florets from *S. squalidus*. Although some *S. cambrensis* were found to be self-sterile (Brennan and Hiscock, 2010). Compared to the tetraploid parent *S. vulgaris*, *S. eboracensis* was also found to have more stigmatic papillae that facilitates pollen capture (Richards, 1986), and higher production of pollen grains which are the main food source of its pollinators (Gilbert, 1986). These changes in reproductive traits (especially self-compatibility) are crucial, especially to new hybrid lineages, to sustain their initial small populations.

### Adaptive Introgression

As reproductive isolation of closely related species is often incomplete, mutations may traverse species boundaries. Low levels of gene flow due to rare interspecific hybridisation may have little effect on neutral diversity within species, but it may be extremely important for genes under positive selection, which can spread across a subdivided ‘population’ (i.e., several hybridising species) with very little gene flow (Slatkin, 1976; Slatkin and Wiehe, 1998). Natural selection may substantially accelerate the transfer of genes between the species (reviewed in Barton, 2001), and horizontal gene transfer (HGT) in bacteria is often detected for genes conferring advantage to their hosts (Ochman et al., 2000; Bapteste et al., 2004), such as antibiotic resistance, or a ‘widespread colonization island’ locus that is involved in adherence and colonisation of surfaces (Planet et al., 2003). The extent and the role of HGT in non-microbial organisms is less clear.

Sharing of adaptive mutations may significantly accelerate adaptation process, as species do not have to ‘wait’ for an adaptive mutation to arise *de novo* (Seehausen, 2004). Sharing of adaptive mutations between species is likely to be particularly important for species with small population sizes, such as endemic adaptive radiations on islands, while species with large population sizes may have sufficient standing variation to make sharing of adaptive mutations unimportant. However, this conjecture remains to be tested. While the number of examples of adaptive gene sharing is growing (e.g., Kapralov and Filatov, 2006; Meier et al., 2017; Richards and Martin, 2017) the role of adaptive allele sharing in adaptation and speciation is still far from clear.

Previous studies have identified multiple cases of cytonuclear phylogenetic discordance (e.g., Shaw, 2002), suggesting introgression of chloroplast or mitochondrial DNA, but cytoplasmic DNA may be particularly prone to interspecific introgression (Tsitrone et al., 2003) and may not reflect the situation with nuclear genes. The literature survey of *F*_st_ values and selection gradients and differentials in phenotypic traits suggested that ‘collective evolution’ of species exchanging adaptive alleles may be fairly widespread (Morjan and Rieseberg, 2004), but more work is needed to clarify the importance of this factor in evolution.

An excellent example of adaptive introgression is the one responsible for adaptation to serpentine soils in *Arabidopsis* (Arnold et al., 2016) and wing colours in mimic *Heliconius* (Pardo-Diaz et al., 2012). Another example of apparently adaptive introgression was also reported for two *Senecio* species in the United Kingdom (Kim et al., 2008). *Senecio vulgaris* that normally does not have ray florets on the capitula, evolved a variety, *S. vulgaris* var. *hibernicus*, which possess rayed capitula like *S. squalidus* following introgression from the latter species (Abbott et al., 1992; Kim et al., 2008). The production of ray florets in this variety of *S. vulgaris* involves the expression of various cycloidea (CYC)-like genes (Kim et al., 2008; Garcês et al., 2016), and was proven to enhance pollination attraction (Abbott and Irwin, 1988) and maternal outcrossing (Marshall and Abbott, 1982, 1984) compared to the non-introgressed *S. vulgaris*. In another pair of *Senecio* species in the Bavarian Forest National Park, Germany, low-elevation *S. ovatus* has benefitted from adaptive introgression from the high-elevation *S. hercynicus*, with introgressed traits related to climatic conditions at high elevations and also shorter vegetative phases as *S. ovatus* spreads towards higher elevations (Bog et al., 2017).

The spread of globally adaptive mutations across several species should result in the loss of species divergence, the loss of intraspecific polymorphism and a characteristic bias in the frequency spectrum of mutations towards rare alleles for the region adjacent to the advantageous gene (Braverman et al., 1995). On the other hand, diversifying selection is expected to reduce gene flow and inflate species differentiation for genes involved in traits that have differing adaptive significance in the two species. Coupled with the effects of adaptive gene sharing, diversifying and adaptive selection are expected to create a mosaic genome, with some parts of the genome having very little divergence between species, while other parts may show strong interspecific differentiation, so called genomic ‘speciation islands’. Such islands were reported in several animal (Duranton et al., 2018; Irwin et al., 2018; Hejase et al., 2020; Zhang et al., 2021) and plant (Renaut et al., 2013; Tavares et al., 2018; Papadopulos et al., 2019) species, including the high- and low-elevation *Senecio* species on Mount Etna, where the genes with high interspecific differentiation clustered around the regions with quantitative trait loci responsible for phenotypic differences between the species (Chapman et al., 2016). However, how much adaptive gene sharing occurs in this *Senecio* hybrid zone remains to be tested.

### Evolution of Novel Compounds

Besides gaining adaptive advantages from mixing parental genomes, hybridisation can also drive the evolution of novel compounds that neither parent can produce, such as secondary metabolites in plants (Rieseberg et al., 1993; Orians, 2000). This is likely due to new selective pressures experienced by the putative hybrids, especially when they occupy novel habitats. Novel compounds can be synthesised by a number of mechanisms, including inhibition or re-direction of biochemical pathways, change in regulatory genes hence gene expression, and segregation of alleles (Orians, 2000). One example is the evolution of a novel methylated luteolin derivatives (flavonoids) in hybrids between *Salix viminalis* and *S. dasyclados*, which are involved in resistance against the lead beetle *Phratora vulgatissima* (Torp et al., 2013).

A novel pyrrolizidine alkaloid, florosenine, that is potentially involved in resistance against thrip species was also discovered in synthetic and natural hybrids between *S. jacobaea* and *S. aquaticus* (Kirk et al., 2010). Although florosenine has been found in other *Senecio* species in other areas (Mendez et al., 1990; Reina et al., 1993; Pelser et al., 2005), it has never been reported for the two species in the studied population and other European popoulations except for one *S. jacobaea* individual with trace amount, likely due to introgression (Kirk et al., 2004, 2010). This suggests the novelty of florosenine in *S. jacobaea* and *S. aquaticus*, although further confirmation is required (Kirk et al., 2010).

### Gene Redundancy

Another potential for evolution lies within redundant genes in the duplicated genomes in auto- and allopolyploids. There are many outstanding questions regarding gene redundancy, such as how it varies among species and its relationship with genome architecture (Barghi et al., 2019). Many duplicated genes are inactivated due to accumulation of mutations (Parisod et al., 2009). They could also be eliminated in the hybrid genomes (e.g., in wheat: Shaked et al., 2001; in maize: Lai et al., 2004; in *Tragopogon miscellus*: Tate et al., 2006). A consequence of sequence elimination is divergence of homoeologous chromosomes preventing their meiotic pairing. Polyploid hybrid lineages have also been shown to purge redundant genomic regions that are far from adaptive optimum as they progress to behave in a more diploidised way (Wu et al., 2006), potentially allowing for better adaptation in novel habitats (Paun et al., 2007).

Nonetheless, there is some empirical evidence hinting on the role of redundant genes in adaptation and speciation. There are many possible reasons why these genes are not purged, for example due to gene balance (Freeling and Thomas, 2006; Birchler and Veitia, 2007) or dosage balance (Conant and Wolfe, 2008). In the early stages of possessing gene redundancy (such as soon after polyploidisation), the hybrid lineage also has a lower chance of creating homozygous recessive genotypes (Comai, 2005). Selection can act on the redundant genes that are not inactivated or purged to diversity gene function (Comai, 2005). They could either evolve new (neofunctionalisation) or complementary functions (subfunctionalisation; Lynch et al., 2001; Parisod et al., 2010). For example, gene redundancy has been suggested to be the basis of polygenic adaptation to new temperature regimes in *Drosophila simulans* (Barghi et al., 2019).

Similarly, in an experiment using *Senecio lautus* it was found that replicate populations of the same ecotype showed parallel evolution of similar phenotypes through different mixtures of adaptive alleles or different mutations in different genes that underlie the same biological functions (James et al., 2021). Most SNPs and genes studied in the divergence between the dune and headland ecotypes were not shared (non-parallel evolution); among all the candidate outlier SNPs, only five were shared across the whole system (James et al., 2021). These indicate that there is plenty of genetic redundancy underlying each biological function in the species (James et al., 2021).

## Concluding Remarks

Hybridisation may not always allow for adaptation and speciation. There is a trade-off between the advantages and disadvantaging of combining divergent genomes. For example, hybrids could obtain the advantageous, higher environmental tolerance, while possessing intermediate traits between the parents that are disadvantageous for surviving in parental habitats (Shimizu-Inatsugi et al., 2017). The successful establishment of hybrids depends on a complex interplay of many evolutionary mechanisms, some of which were discussed in this article. The research in the genus *Senecio*, especially the work focused on the *S. aethnensis*—*S. chrysanthemifolius*—*S. squalidus* system, has significantly advanced our understanding of adaptation and speciation. In particular, the work in this system revealed some of the roles hybridisation could play in evolution, including transcriptome shock (e.g., up-regulation of genes linked to sulphur deficiency), genome reorganisation (e.g., between *S. aethnensis* and *S. chrysanthemifolius*, and inherited in *S. squalidus*), change in mating system and reproductive traits (e.g., self-compatibility and gain of ray florets in *S. cambrenisis* and *S. eboracensis*, hybrid species involving self-incompatible *S. squalidus* and self-compatible *S. vulgaris*), and adaptive introgression (e.g., gain in ray florets in *S. vulgaris* through hybridisation with *S. squalidus*). Other aspects, such as evolution of novel compounds, gene redundancy, and the extent of adaptive allele sharing, have been explored in other *Senecio* species (e.g., novel florosenine in *S. jacobaea*, heterosis in *S. jacobaea x S. aquaticus* hybrids), but remain to be explored in the *S. aethnensis*—*S. chrysanthemifolius*—*S. squalidus* system. This showcases the research potential of *Senecio* as a whole to not only study the role of hybridisation in speciation and adaptation, but also other questions in evolutionary biology and ecology (reviewed in Walter et al., 2020). With the worldwide distribution of a vast number of species and ease of cultivation, *Senecio* offers great potential for evolutionary biologists to address outstanding questions regarding the role of hybridisation in adaptation and speciation. Specifically, how do hybridising species maintain their identity despite their gene pools being homogenised by hybridisation and interspecific gene flow? How do hybridising (sub)species diverge and evolve reproductive isolation? How strong and widespread in the genome diversifying selection should be to drive speciation of actively hybridising (sub)species? Under what conditions (e.g., large versus small populations) interspecific hybridisation plays more important in adaptation and speciation processes? The upcoming *S. squalidus* genome will help to address these questions using the *S. aethnensis*—*S. chrysanthemifolius*—*S. squalidus* system.

## Author Contributions

ELYW and DAF came up with the concept of this mini-review. ELYW wrote the initial draft and all authors contributed to editing. All authors contributed to the article and approved the submitted version.

## Funding

This work was funded by NERC (NE/P002145/1) projects to SJH and DAF as well as by a BBSRC grant (BB/P009808/1) to DAF.

## Conflict of Interest

The authors declare that the research was conducted in the absence of any commercial or financial relationships that could be construed as a potential conflict of interest.

## Publisher’s Note

All claims expressed in this article are solely those of the authors and do not necessarily represent those of their affiliated organizations, or those of the publisher, the editors and the reviewers. Any product that may be evaluated in this article, or claim that may be made by its manufacturer, is not guaranteed or endorsed by the publisher.
